# Focus on advanced nanoprocessing and applications in sensorics

**DOI:** 10.1080/14686996.2017.1368257

**Published:** 2017-09-15

**Authors:** Maria Bardosova, Hiroshi Fudouzi

**Affiliations:** ^a^ Micro & Nano Systems Centre, Tyndall National Institute, University College Cork, Cork, Ireland; ^b^ Faculty of Electrical Engineering and Information Technology, Slovak Technical University in Bratislava (STUBA), Bratislava, Slovak Republic; ^c^ Research Center for Functional Materials, National Institute for Materials Science, Sengen, Tsukuba, Japan

Contemporary science is, on the one hand, becoming more and more specialized and deals with smaller and smaller objects. On the other hand, it studies many interesting phenomena that occur at the borders between two or more ‘traditional’ scientific fields. This is especially true in the case of materials science, which fuses different parts of physics, chemistry and biology, and whose results require *nanoprocessing* technologies in order to manufacture novel devices that outperform the existing ones, present less burden to the environment, conserve natural resources and support sustainability. Many such devices can operate interactively and autonomously thanks to electronics, which receive feedback from the physical environment via sensors. *Sensorics* deals with design, manufacture and the processing of signals from sensors. It is a rapidly evolving research area owing to the abundance of physical properties it is necessary to deal with in the newly developed devices, a number on par with the phenomena and materials available to do so.

This Focus Issue includes seven selected papers with subjects related to nanoprocessing and sensorics that were presented at the nanoMA 2015 conference. The meeting was devised by members of the consortium working on a Framework 7 (FP7) grant project titled ‘Photonic Applications of Nanoparticle Assemblies and Systems’ (PhANTASY) within the Marie Curie International Research Exchange Scheme (IRSES).

Schopf et al. [1] used individual Au nanorods immobilized on solid substrates as plasmonic transducers to investigate the deposition of mercury towards the development of a miniaturized mercury plasmonic sensing system.

Khan and Corbett [2] showed that Bloch surface waves (BSW) resonances can be used in label-free sensors providing larger sensitivities and lower detection limits than metal-based surface plasmon resonance sensors and proposed a waveguide-coupled BSW suitable for on-chip sensing.

Majima et al. [3] fabricated a chemically assembled single-electron transistor with a passivation bilayer comprising an alkanethiol/alkanedithiol self-assembled monolayer and pulsed laser deposited aluminium oxide (AlO_x_). This combination of bottom-up and top-down processes enables the connection of CMOS technologies via planar processes.

Zhou and Azumi [4] reviewed the possibilities and challenges of using carbon nanotubes (CNT) as transparent conductive films (TCFs). Industrial applications in this field are currently dominated by indium tin oxide (ITO), while alternative materials include metal oxides, conducting polymers and metal nanowires and grids. The authors focus on recent progress in the fabrication and modification of CNT-based TCFs, discuss their properties and stability and outline the challenges to be addressed: high production costs and insufficient conductivity and transparency.

Collins et al. [5] reviewed recent advances in inverse opal structures and their applications with a focus on their use as catalysts, catalyst support materials, electrodes for batteries, water-splitting photoanodes, solar-to-fuel converters and electrochromic devices.

Shrestha et al. [6] prepared high surface area nanoporous carbon materials (NCM) from bamboo cane by chemical activation using phosphoric acid. The resulting materials were characterized by various techniques and their surface area and pore volumes studied. The authors discuss the possible use of NCM in energy storage devices and for vapour sensing of volatile organic solvents.

Ryan et al. [7] prepared a range of cross-linked chitosan composites, functionalized with silver and gold, and studied their structural and mechanical properties. Chitosan and silver and gold nanoparticles are well known individually for their inherent antimicrobial properties. The aim of the study was to investigate whether combining these materials would lead to any enhanced antimicrobial effects.

The nanoMA 2015 conference was organized jointly by the National Institute for Materials Science (NIMS), Tsukuba, Japan, and Tyndall National Institute UCC Cork, Ireland. Both institutes are national flagships that carry out fundamental research in materials science using nanotechnology and deliver innovative solutions for industrial applications.
